# Contribution of *In Vivo* and Organotypic 3D Models to Understanding the Role of Macrophages and Neutrophils in the Pathogenesis of Psoriasis

**DOI:** 10.1155/2017/7215072

**Published:** 2017-11-08

**Authors:** Isabelle Lorthois, Daniel Asselineau, Nathalie Seyler, Roxane Pouliot

**Affiliations:** ^1^Centre LOEX de l'Université Laval, Génie Tissulaire et Régénération, Centre de Recherche FRQS du CHU de Québec, Axe Médecine Régénératrice, Québec, QC, Canada; ^2^Faculté de Pharmacie, Université Laval, Québec, QC, Canada; ^3^L'Oréal Research & Innovation, Aulnay-sous-Bois, France; ^4^Episkin Academy, Lyon, France

## Abstract

Psoriasis, a common chronic immune-mediated skin disease, is histologically characterized by a rapid keratinocyte turnover and differentiation defects. Key insights favor the idea that T cells are not the only key actors involved in the inflammatory process. Innate immune cells, more precisely neutrophils and macrophages, provide specific signals involved in the initiation and the maintenance of the pathogenesis. Current data from animal models and, to a lesser extent, three-dimensional *in vitro* models have confirmed the interest in leaning towards other immune cell types as a potential new cellular target for the treatment of the disease. Although these models do not mimic the complex phenotype nor all human features of psoriasis, their development is necessary and essential to better understand reciprocal interactions between skin cells and innate immune cells and to emphasize the crucial importance of the local lesional microenvironment. In this review, through the use of *in vivo* and 3D organotypic models, we aim to shed light on the crosstalk between epithelial and immune components and to discuss the role of secreted inflammatory molecules in the development of this chronic skin disease.

## 1. Introduction

Psoriasis is a chronic autoimmune disease that affects 2-3% of the world's population, characterized by hyperproliferation and abnormal differentiation of epidermal keratinocytes [1–6]. Psoriatic skin lesions are also characterized by increased permeability of lymphatic capillaries, increased blood flow, and angioproliferation [7–10]. Eighty percent of patients suffer from mild to moderate forms of the disease, while 20% of patients develop moderate to severe psoriasis, affecting more than 5% of their body surface area [11]. It is also known that patients with plaque psoriasis have an increased risk of inflammatory diseases affecting noncutaneous sites—including psoriatic arthritis, cardiovascular disease, and inflammatory bowel disease—associated with common pathophysiological mechanisms. These comorbidities are multifactorial and in many cases related to inflammation, induced by close pathogenic mechanisms related to cytokine dysregulation.

PSORS1 is the major susceptibility locus for psoriasis vulgaris and lies within an approximately 300 kb segment of the major histocompatibility complex on chromosome 6p21.3 [12–14]. Several studies have indicated *HLA-Cw6* as the primary *PSORS1* risk allele within the candidate region, coherent with the fact that MHC class I molecules play an important role in the function of CD8^+^ T cells [15, 16].

More than 32 PSORS have been identified, containing genes involved in inflammatory metabolic pathways and epidermal proliferation as well as skin barrier function, but have not demonstrated their complete involvement in pathology. Also, the variations in the number of copies of a gene may be involved in the pathology. For example, beta defensins, antimicrobial peptides involved in innate immunity, are a good example of a gene known to be associated with psoriasis. Of the 8 defensins, the hBD-2, hBD-3, and hBD-4 proteins encoded, respectively, by DEFB2, DEFB3, and DEFB4 were linked to keratinocyte stimulation via proinflammatory interleukins 8, 18, and 20 [17]. The eight defensin genes are linked on two different chromosomes, chromosome 20 as well as chromosome 8p23.1. Most of the defensin genes encoded on chromosome 8p23.1 have longer gene repeat units, which are highly variable in copy number. Several studies have attributed a relationship between psoriasis and the number of gene copies of these defensins [18]. In 2012, a meta-GWAS (genome-wide association studies), which aims to identify SNPs (single nucleotide polymorphisms) in DNA associated with a clinically defined disease (phenotype) by comparing the allele frequency of each SNP between a group of individuals with psoriasis versus healthy patients, confirmed 21 SNPs, and identified 15 new SNPs [19].

The current research tends to demonstrate that the process is initiated by an inflammatory immune reaction against autoantigens of the skin, in which dendritic cells, T lymphocytes, macrophages, and neutrophils play a pivotal role. Dendritic cells, antigen-presenting cells, are present in greater numbers in psoriatic lesions. Dendritic cells of lymphoid origin, such as plasmacytoid dendritic cells, would be involved in initiating lesions [20], recognizing autoantigens, and causing IFN-*α* secretion by these cells [21]. This would follow the activation of innate immunity cells, such as neutrophils or macrophages, and adaptive immune cells, such as T lymphocytes. Persistent activation of these cells would lead to the chronicization of psoriatic lesions, such as a vicious circle of inflammation [2, 22, 23].

Resident macrophages and dendritic cells are among the cells most involved in the “sensing” of danger signals. The activation of macrophages via the secretion of proinflammatory cytokines, such as IL-6 and TNF-*α*, but also of chemokines, such as CXCL8 (also known as IL-8), CCL5, CXCL1, and CXCL2, promotes the recruitment of inflammatory cells, like neutrophils [24]. Multiple signals are likely to trigger, via interaction with their receptor(s), a secretion of chemokines, thus attracting neutrophils to the inflammatory site. Macrophages and neutrophils may act as T lymphocyte-dependent effectors, as they are present at the site of inflammation even before a specific immune response has developed.

It is therefore evident that monocytes, macrophages, and neutrophils have a particular function in the early phases of inflammation, and their role in driving and maintaining this inflammatory process in the pathogenesis of psoriasis must be clarified. Here, we aim to discuss the specific role of innate immune cells, such as neutrophils and macrophages, in the initiation and the sustainability of chronic inflammatory skin diseases, such as psoriasis, through the use of organotypic models and mouse models. These models allow a better understanding of cellular and molecular mechanisms with the aim of identifying new potential therapeutic targets.

## 2. Macrophages

**Figure 1:**
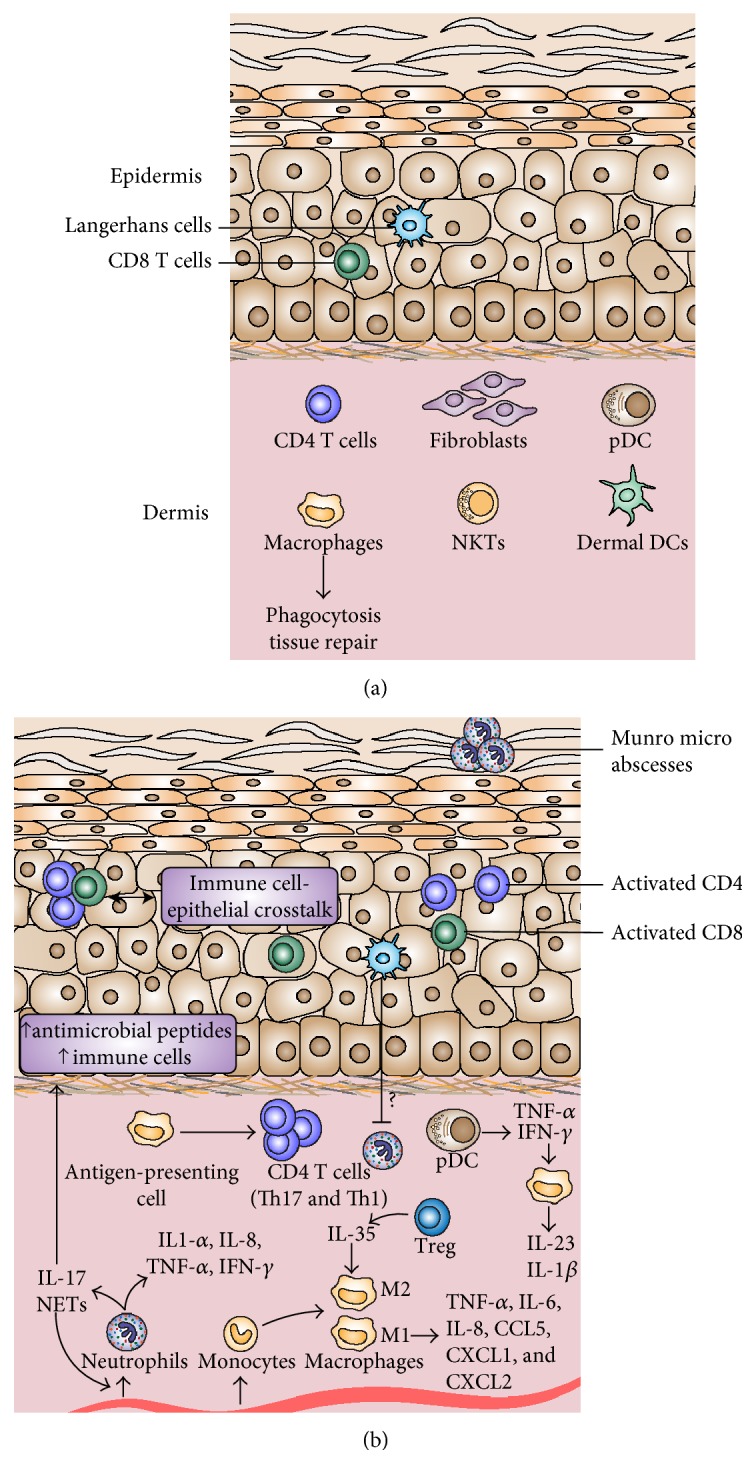
Location and function of macrophages and neutrophils in healthy (a) and psoriatic (b) skin. Langerhans cells and CD8 T cells are present in healthy epidermis, while CD4 T cells, as well as macrophages, NK T cells, dermal dendritic cells (DCs), and plasmacytoid dendritic cells (pDCs), are found in the dermis mainly composed by fibroblasts and extracellular matrix. Immune cell infiltrates are present in the psoriatic epidermis, consisting of activated CD4 and CD8 T cells and clusters of Munro's abscess in the *stratum corneum*. In the dermis, the activation of cells of both innate and adaptive promotes an inflammatory response. Neutrophils and macrophages (differentiated from monocytes) infiltrate the dermis and secrete proinflammatory cytokines. The crosstalk between skin cells and immune cells leads to a complex inflammatory response and contributes to the development of a pathological epithelial phenotype. NET: neutrophil extracellular trap; Treg: regulatory T cell; M: macrophage.

Monocytes can differentiate to become tissue-resident macrophages or dendritic cells. Macrophages are phagocytic cells [25] within the dermis, important for tissue homeostasis and the regulation of lymphocyte activation and proliferation [26]. Some macrophages are long-term tissue residents and play an important role in controlling the repair [27, 28] and regeneration of skin tissue [29]. Inflammatory macrophages, on the other hand, participate in the innate immune response and play a dual role in the immune system as phagocytes and antigen-presenting cells capable of activating T lymphocytes (Figure 1).

As early as the 1980s, some evidence of increased macrophage activity in psoriasis [30] highlights their key role in inducing psoriasis-like skin disease.

In 2010, Fuentes-Duculan and colleagues observed that a subpopulation of CD163-positive macrophages was found mostly in psoriatic lesions. The CD163 marker, a scavenger receptor intervening in the elimination of the hemoglobin-haptoglobin complex, expressed both on the surface of the mature tissue macrophages and on blood monocytes, is in normal human skin, a marker more assimilated to the so-called “alternative” macrophages or M2 [31]. However, CD163-positive macrophages also express IFN-*γ*-regulated genes (STAT1, CXCL9), and IFN-*γ* is known to be a “type 1 cytokine.” These results suggest a great phenotypic plasticity of the macrophages in responding to their environment and acquiring new properties and new markers, while preserving their original characteristics.

Other macrophage markers such as RFD7 [32], CD68 [33, 34], CD107 [35, 36], MARCO [37], Stabilin-1 [38], and MS-1 [39, 40] are not generally expressed at the same time on their surface, but their expression fluctuates according to their location in the skin compartments. For example, CD68^+^ cells coexpress CD163 in the upper reticular dermis while they do not colocalize with CD163 near the dermoepidermal junction. It has also been shown that these markers coexpress to some extent CD11c, a marker of myeloid dendritic cells, but CD163 has the weakest coexpression with CD11c, an ideal candidate for labeling macrophages in psoriasis [31]. The cutaneous macrophages of lesional plaques probably do not have a single phenotype, M1, or M2 but rather have a mixed, microenvironment-dependent phenotype, associated with the different roles they can play in a context of chronic inflammation.

Egawa et al. have also demonstrated that CCR2^+^ monocytes recruited at inflammatory sites had the potential to acquire an M2 phenotype in response to IL-4, thus exerting an anti-inflammatory function [41], while the expression of CCR2 (MCP-1/CCL2 protein receptor) of the peripheral monocytes of patients with psoriasis or atopic dermatitis was increased compared to that of healthy patients [42]. It has also been demonstrated that macrophages M1 or M2 have the ability to be repolarized by the cytokines of Th2 or Th1 lymphocytes, respectively [43].

The hypomorphic PL/J CD18 murine model is characterized by reduced expression (2–16% of the wild-type levels) of the common integrin 2 (CD11/CD18) chain, leukocyte adhesion molecules required for cell-cell contacts. Transgenic mice generally develop psoriasiform skin inflammation: erythema, scaling, abnormal keratinocyte proliferation/differentiation, subcorneal microabscesses, and increased inflammatory infiltrate. The authors reported that the activation of cutaneous macrophages by recombinant MCP-1 or LPS alone was not sufficient to produce chronic psoriasis-like inflammation as observed *in vivo* in human skin lesions [44]. Nevertheless, the combined injection of recombinant murine MCP-1 and TNF-*α* in nonlesional skin area of hypomorphic CD18 mice led, respectively, to the accumulation and activation of macrophages, unlike CD4-positive T cells. Activated macrophages would secrete more TNF-*α*, responsible for sustained activation of macrophages, thus causing a positive feedback loop. T cells, mast cells, and endothelial cells would participate directly in the chronicization of lesions via TNF-*α* secretion. Finally, depletion of skin macrophages in this murine model via the injection of clodronate liposomes (specific depletion of macrophages) and the neutralization of a single cytokine, TNF-*α*, attenuates the severity of skin inflammation, emphasizing the importance of macrophages in the psoriasis physiopathology.

A specific deletion of the keratinocyte NF-*κ*B kinase inhibitor (IKK2) in mice results in an inflammatory and hyperproliferative cutaneous phenotype. The treatment of transgenic mice with a TNF-neutralizing antibody abolished the inflammatory phenotype and therefore improved the skin phenotype. The injection of clodronate liposomes in this murine model restored the expression of early and late keratinocyte differentiation markers and reduced the number of granulocytes and T cells present, highlighting the importance of macrophages in the accumulation of granulocytes and T cells in inflammatory skin areas [45]. Moreover, a K14-Cre-IKK2^fl/fl^ murine model showed the upward expression of gene coding for proteins regulated by IFN-*γ*. The presence of IFN-*γ* receptor may accelerate the onset of the psoriasis-like inflammatory skin disease in K14-Cre-IKK2^fl/fl^ mice but is not essential for it to develop. The authors demonstrated that the migration of macrophages to the lesional skin areas and their subsequent activation was a necessary key feature for the development of psoriatic inflammation. The activated macrophages were then able to initiate and maintain psoriasiform skin inflammation. These investigations were also commented in the *Journal of Clinical Investigation* by Clark and Kupper in 2006 [46].

The topical application model of imiquimod (IMQ), a 7/8 TLR (toll-like receptor) agonist widely used in mice, activated immune cells, such as macrophages and plasmacytoid dendritic cells. The mice displayed a hyperplastic cutaneous epithelial-squamous phenotype similar to human psoriasis. Imiquimod-treated mice, KO for CX3CR1 (fractalkine/CX3CL1 receptor), developed minor inflammation compared to WT mice. In fact, the production of IL-12, IL-23, IL-17A, IL-22, IL-6, IL-1, TNF-*α*, and IL-36 cytokines was decreased in these mice. The macrophages of CX3CR1^−/−^ mice expressed CCR2, unlike WT mice treated with IMQ, and attenuated the inflammation generated, in part, by Th1/Th17 lymphocytes, following functional changes [47]. The authors hypothesized that CCR2 could partially compensate for the loss of CX3CR1 by directing the migration of resident macrophages. Surprisingly, CCR2^−/−^ mice exhibited an exacerbation of inflammation despite altered recruitment of inflammatory monocytes to cutaneous sites. CX3CR1^−/−^ mice expressed fewer M1 macrophage markers compared to WT mice, suggesting that decreasing the number of M1 macrophages would contribute to decreased inflammation resulting from CX3CR1 deficiency. Finally, a transfer of macrophages from WT mice to CX3CR1^−/−^ mice normalized psoriasiform type inflammation induced by IMQ, emphasizing the importance of macrophages in the regulation of psoriatic inflammation.

The KC-Tie2-overexpressing mice developed a cutaneous psoriasiform phenotype. These mice spontaneously developed characteristic hallmarks of human psoriasis, including acanthosis (hyperplasia of the epidermis), increases in dermal CD4^+^ T cells, infiltrating epidermal CD8^+^ T cells, dermal dendritic cells and macrophages, and increased expression of cytokines and chemokines associated with psoriasis (IFN-*γ*, TNF-*α*, IL-1*α*, IL-6, IL-12, IL-22, IL-23, and IL-17). Cathelicidin, *β*-defensin, and S100A8/A9 were also upregulated in the hyperproliferative skin [48].

The administration of clodronate liposomes in the skin of transgenic KC-Tie2 mice resulted in the elimination of F4/80^+^ macrophages, CD11b^+^ myeloid cells, and CD11C^+^ dendritic cells. The eradication of these cells would result in the disappearance of acanthosis, a decrease in the number of T cells, and a significant reduction in the production of TNF-*α*, IL-23, IL-1, IL-6, and S1008/9, stressing the importance of myeloid cells and their cytokines in maintaining the pathology [49].

The use of murine models, in particular the topical application of imiquimod in mice, makes it possible to mimic the dominance of monocytes, neutrophils, and dendritic cells derived from monocytes at an early lesion stage, and later, the decreased number of neutrophils and monocytes and the parallel increase in the number of dermal macrophages and Langerhans cells [50], which are then depleted. Langerhans cells are found mainly in the spinous layer of the epidermis. It is estimated that they constitute 2 to 4% of the epidermal cell population [51]. Derived from the bone marrow, they are transported by the blood to the epidermis [52]. After capturing the outer antigens, they migrate to the lymph nodes where they will initiate a specific immune response by displaying these antigens to the T lymphocytes. Langerhans cells are therefore antigen-presenting cells [52]. Similarly, in the murine DKO model, whose Jun and JunB transcription factors were deleted, resulting in psoriasiform type inflammation, the increase in the number of Langerhans cells (LCs) by proliferation followed by their subsequent decrease would reproduce the presence of proliferative Langerhans cells in a human lesional context [53]. In this model, the authors indicated that LCs would exert an immunoregulatory role by increasing the expression of IL-10 and PD-L1. Without LCs, the absence of regulatory signals would result in increased skin inflammation in these mice. The depletion of LCs did not alter the number of regulatory T cells in the skin, thus excluding the possibility that a reduced number of regulatory T cells could be responsible for worsening of the pathology. In addition, genetic depletion of LCs during the inflammatory phase in mice treated with imiquimod caused increased neutrophil infiltration and extension of pustular plaques, suggesting an anti-inflammatory role for LCs during psoriatic inflammation [50].

Recently, Leite Dantas et al. focused on the contribution of macrophages and T cells in the development of psoriasiform inflammation in a transgenic mouse model ihTNFtg (doxycycline- [Dox-] inducible human TNF-transgenic mouse line). In this murine model, the authors found that only macrophages (M1 and M2), Th1, and Treg were present in large quantities. While depletion of macrophages greatly reduced the development of the disease, Treg depletion increased the infiltration of macrophages into psoriatic inflammatory areas, contributing to the worsening of the pathology. Adoptive transfer of Treg in RAG-1-deficient mice, without either mature B or T lymphocytes, or immunocompetent mice induced the opposite effect, attenuation of symptoms. Thus, Tregs would limit migration of macrophages to injured areas, thereby reducing the harmful effect on tissue of macrophages in these transgenic mice [54].

Recently, IL-35, produced by regulatory T cells, demonstrated immunosuppressive effects in mouse models of psoriasis. Indeed, IL-35 may reduce the local infiltration of macrophages by reducing the levels of cutaneous expression of the macrophages M1 while conversely increasing M2 macrophages. Also, IL-35 may regulate the production of proinflammatory CD4^+^ T cell cytokines and may decrease local lymphocytic infiltration of Th17 cells in K14-VEGF-A-Tg mice and in mouse models of imiquimod-induced psoriasis [55]. IL-37, expressed in macrophages, epithelial cells, and effector-memory cells, likewise demonstrated an immunosuppressive role in K14-VEGF-A transgenic mice by downregulating the production of proinflammatory cytokines, such as CXCL8, IL-6, and S100A7 [56]. IL-37 acted as a negative feedback inhibitor of inflammatory responses as the reduction of IL-37 protein synthesis in PBMCs with specific siRNA increased the production of several proinflammatory mediators [57] (Table 1).

## 3. Neutrophils

Polymorphonuclear neutrophils (PMNs), or leukocytes, are phagocytic cells characterized by a segmented lobular nucleus and cytoplasmic granules filled with degradation enzymes. PMNs are the most abundant circulating white blood cells and are the first type of cells involved in acute inflammatory reactions to bacterial infections [58]. These phagocytes ingest the microbes and release reactive oxygen species, antimicrobial peptides, proteases, and neutrophil extracellular traps [59, 60].

Neutrophils infiltrate psoriatic lesions early from blood vessels within the dermis and form microabscesses, called Munro abscesses in humans, due to their accumulation in the form of microbial clusters in the thickened and parakeratotic stratum corneum [61] (Figure 1). Neutrophils accumulate in the skin, attracted by a gradient of chemotactic factors, which may be small induced secreted cytokines, such as IL-8, NAP-2, and NAP-3; membrane lipid derivatives such as leukotriene and platelet-activating factor (PAF); or substances of bacterial origin (LPS) [62–65].

Schon et al. noted that neutrophil depletion in the flaky skin (fsn)/fsn mutant mouse model of psoriasis-like lesion development contributed to a decrease in epidermal thickness, neutrophilic infiltrate, epidermal microabscesses, and the number of CD3-positive T cells, thus to an improvement of psoriasiform skin lesions [61].

Extravasation of neutrophils was made possible by the binding of integrin *α*_M_*β*_2_ (CD11b/CD18) to ICAM-1 (CD54) of endothelial cells. ICAM-1 would be expressed de novo on the surface of hyperproliferative psoriatic keratinocytes, thus contributing to the migration of neutrophils expressing *α*_M_*β*_2_ to the epidermis [61].

Some neutrophil-derived T cell attractants, such as *defensin-1*, *defensin-2*, or CAP37/azurocidin [66], may explain the reduction in the number of tissue T lymphocytes upon *in vivo* depletion of neutrophils in SCID mice. However, the influx of neutrophils into the epidermal compartment via chemoattractants seems to follow the influx of lymphocytes.

Moreover, the depletion of neutrophils in patients with moderate to severe generalized pustular psoriasis via an extracorporeal circulation therapy that selectively eliminates elevated myeloid lineage leukocytes resulted in a decrease in erythroderma, pustules, and edema up to 10 weeks after therapy [67].

Neutrophils would represent a major source of IL-17 [68–70], via the formation of extracellular traps [71], whose production is defined as the ultimate stage in a process of neutrophil polymorphonuclear activation. The topical application of leukotriene B4 (LTB4), found in high concentrations in psoriatic lesions, is a chemoattractant for neutrophils, eosinophils, monocytes, macrophages, mast cells, dendritic cells, and effector T cells. LTB4 induced a rapid influx of polymorphonuclear cells into the epidermis and dermis, followed by an infiltrate of mononuclear cells [69]. Moreover, a recent study has demonstrated that LTB4 receptor 1 (BLT1) and CXCR2 promoted the recruitment of neutrophils at psoriatic lesional sites and that these cells would secrete IL-1*β*, perpetuating psoriatic inflammation [72] (Table 1).

Moreover, the release of this mediator, IL-17, by NETosis amplified the accumulation of neutrophils [73] by increasing the expression of CXCL1, CXCL2, and IL-8. IL-17 increased the expression of antimicrobial peptides—*β*-defensin-2 (HBD-2), S100A7, S100A8, S100A9, and LL37—by keratinocytes [74–76]. These antimicrobial peptides can stimulate immune cell infiltration, and NET-derived DNA-LL37 nucleic acid complexes promoted IFN-*α* secretion of plasmacytoid dendritic cells [21]. IFN-*α* and TNF-*α* would stimulate the influx of inflammatory dendritic cells and macrophages, which would produce cytokines, including IL-23 and IL-1*β*, in the presence of IFN-*γ*.

Blocking IL-17A via a neutralizing antibody (secukinumab) reduced hyperkeratosis, acanthosis, and hyperproliferation, significantly decreased the levels of gene expression of chemokines derived from keratinocytes, such as CXCL1 (GRO) and CXCL8 (IL-8), and triggered near-total elimination of IL-17-positive epidermal neutrophils. The authors suggested that the inhibition of IL-17A would indirectly block the influx of neutrophils due to the lack of keratinocyte response to IL-17A [70].

Furthermore, the specific deletion of the A-chain of IL-17 receptor in mice contributed to the delay and attenuation of psoriatic inflammation in mice treated with imiquimod but did not prevent its development. KO mice for IL-17RA showed a delay and alteration of peripheral neutrophils at the site of injury. The authors hypothesized that IL-6, strongly expressed in KO mice for IL-17RA and treated with imiquimod, may play a role in the development of pathology in the absence of IL-17RA [77]. Nevertheless, there is no doubt that compensatory mechanisms ensure the attraction of neutrophils to the inflammatory site in the absence of the IL-17RA signaling pathway.

The importance of IL-6 has also been demonstrated in a murine model in which IL-17A and GFP are coexpressed in keratinocytes, resulting in the formation of psoriatic-like lesions. A blockage of IL-6 signaling would reduce the pathology in these mice by reducing the formation of neutrophil microabscesses in the epidermis and reducing the number of myeloperoxidase-positive cells [78].

In 2011, Garcia-Romo et al. reported that NETs would produce a greater amount of LL37 in response to PMA and IFN-*γ* in systemic lupus erythematosus [79]. The secretion of LL37 facilitated the uptake and recognition of DNA by plasmacytoid dendritic cells. The antimicrobial peptide LL37 was also overexpressed in the lesional psoriatic skins and would participate in the activation of cells of innate immunity [80].

Neutrophils participate in the secretion of inflammatory mediators, including IL-1*α*, IL-8, TNF-*α*, and IFN-*γ* cytokines [81–84]. The interaction of neutrophils and fibroblasts may increase the secretion of IL-8 [85]. In addition, IL-12, expressed on the surface of mononuclear cells, such as epidermal neutrophils, is overexpressed in psoriatic lesions. Although fibroblasts do not secrete IL-12, the fibroblast-neutrophil interaction upregulates IL-12 secretion, highlighting the importance and necessity of considering cooperation between the different cells to better understand these interactions. Furthermore, IL-12 promoted the survival and growth of Th1 cells, as well as their differentiation, and inhibited the formation of Th2 cells.

The formation of neutrophil-containing microabscesses would be dependent on IL-1R1. It has been demonstrated in the imiquimod-induced murine model that the signaling of IL-1 via IL-1R1 regulated constitutive and induced chemokine expression in response to imiquimod, involved in the *in vivo* recruitment of neutrophils. However, the deletion of IL-1R1 did not block the formation of microabscesses, implying that other cytokines are involved in their formation [86]. CEACAM-1 expression in superficial keratinocytes found in psoriatic lesions would also contribute to the persistence of neutrophils and to the underlying inflammation in psoriatic patients [87].

Aldara cream modifies the immune response by stimulating the body's defenses that fight certain types of skin affections. The topical application of Aldara in mice deficient in IL-17A, IL-17F, or IL-22 drastically reduced the severity of psoriasis. However, *γδ* T cell populations and innate ROR*γ*t-positive lymphocytes produced large amounts of these inflammatory cytokines and were necessary and sufficient for the formation of psoriatic lesion plaques in this murine model. A reverse ROR*γ*t agonist, developed by Janssen, has demonstrated its efficacy in murine models for psoriasis and inflammatory arthritis. The blockade of the Th17 differentiation led to the decrease of the production of IL-17A by the memory T cells and reduced the production of IL-17A and IL-22 by the cells NKT and *γδ* [88].

Furthermore, the combined action of mannan-activated macrophages and IL-17A from T cells provoked the infiltration of neutrophils into skin compartments, leading to histopathological features [89].

IL-17F would induce the secretion of IL-8 by the keratinocytes and would favor the infiltration of neutrophils into the dermis. On the other hand, the blocking of neutrophil infiltration by an anti-IL-8 antibody underlined the importance of the IL-17F/IL-8 axis in the pathophysiology of psoriasis [90].

At last, a treatment with ustekinumab (human monoclonal antibody directed against IL-12 and IL-23p40) or infliximab (monoclonal chimeric antibody directed against TNF), both used to treat psoriasis, in severe psoriatic patients appeared to decrease the activity of neutrophils and monocytes. Indeed, the expression of CD62L, a molecule of cell adhesion, is restored in patients receiving biological therapy, while expressions of CD11b (also called integrin alpha M) and CD66b, another adhesion molecule, were decreased after treatment. Also, the ratio of activated CD14^high^ monocytes was normalized in patients receiving therapy [91], stressing once again the importance of these immune cells in psoriasis.

*In vivo* models have demonstrated the complex but evident interrelationship between different immune cells at the level of psoriatic lesions and provide a better understanding of the influence that cells have on each other and the possible modulating effect of cytokines and chemokines on the functioning of neighboring cells in the local microenvironment (Figure 1).

## 4. 3D Organotypic Skin Models

At present, few organotypic models emphasize the importance of macrophages or neutrophils in psoriasis, but the enthusiasm for such models could lead us to make new discoveries in the years to come.

Some models already demonstrated the role of T cells in the pathogenesis of psoriasis. In the 1990s, transplants of human psoriatic skins in immunodeficient mice [92] and injected with autologous T cells, from either peripheral blood or the lesion site, indicated that only the latter was able to maintain the psoriatic phenotype in the grafted mice [93]. In 2010, to counter the limitations associated with such models, Guerrero-Aspizua et al. isolated both skin cells—keratinocytes and fibroblasts—and peripheral blood from psoriatic patients and reproduced cutaneous equivalents by bioengineering [94]. These equivalents were then grafted onto immunodeficient mice. The authors indicated that intraepidermal injection of activated human immunocytes induced the formation of psoriatic lesion in the skin model of xenotransplantation. The authors observed that the combination of factors secreted by Th1 cells and cytokines derived from Th17 cells was essential for the complete development of a psoriatic phenotype, emphasizing the importance of T cells in the pathology.

In 2002, Del Rio and colleagues performed a long-term follow-up of gene-transferred bioengineered artificial human skin based on a fibroblast-containing fibrin dermal substrate orthotopically grafted onto mice [95]. This preclinical approach, considered more clinically relevant and better predictive models of drug efficacy, will certainly identify new therapeutic targets for psoriasis. Moreover, the topical application of nanosomes containing siRNAs inhibiting the expression of hBD-2 in such a mouse model improved the cutaneous phenotype and reduced the number and the size of blood vessels in the dermal compartment [96].

Modeling psoriatic inflammation requires paying close attention to the immune component. Some organotypic 3D models, with no immune component, attempt to mimic psoriatic inflammation. In our lab, we have already demonstrated that the generation of skin equivalents from human psoriatic fibroblasts and keratinocytes produced by the self-assembly method displayed major hallmarks of psoriasis [97]. Also, although the addition of a cytokine cocktail [94, 98–100], or a single protein [101], to mimic psoriatic inflammation has demonstrated some histopathological aspects of psoriasis, it is unlikely that all mediators released by immune cells will be generated with a mixture of a few inflammatory cytokines. Although essential, animal models cannot reflect the etiology of psoriasis or represent the human complexity associated with pathology.

An alternative to animal experimentation is the development of equivalent three-dimensional models generated from human skin cells. These organotypic models, which are rapidly expanding, make it possible, by adding one or more cutaneous components, to dissect the specific role of each cell type present. Conversely, current models, usually with only one immune component, cannot summarize all the cellular and molecular interactions occurring *in vivo* due to the absence of other components, which are certainly important to the general pathophysiology of psoriasis. Moreover, deletion of an element (macrophages by clodronate liposomes, e.g.) very rarely results in a complete reversion of the phenotype, thus implying that other constituents are involved in the pathology development.

Thus far, too few *in vitro* three-dimensional models including the main cellular components involved in the pathophysiology of autoimmune diseases, such as psoriasis or atopic dermatitis, are currently being studied to further understand the role of the microenvironment in maintaining inflammation. The influence of the components on the modulation of the soluble and nonsoluble factors constituting the local lesional microenvironment can be apprehended by the generation of 3D models. However, the difficulty of incorporating immune cells into skin models, along with the complexity involved in modeling a three-dimensional environment necessary and sufficient to maintain cell viability and to respect the anatomical arrangement of cells, represents a major challenge.

In 2006, Dezutter-Dambuyant et al. optimized the development of a reconstructed skin model by incorporating hematopoietic progenitor cells in an endothelialized skin equivalent. The team demonstrated that the differentiation of dendritic cell precursors into Langerhans cells depends on the state of differentiation of keratinocyte cells. In this case, the differentiation program of the Langerhans cells started only if keratinocytes were differentiated. Interaction between fibroblasts and keratinocytes in a three-dimensional skin model supported the regulation of their own differentiation and also that of anatomically close cells [102].

The encapsulation of dendritic cells in an agarose and fibronectin gel, compartmentalized between a layer of fibroblasts and keratinocytes and treated with dichlorobenzene for 24 hours, makes it possible to study the cellular interactions and mechanisms of skin sensitization [103]. In addition to maintaining their viability and horizontal and vertical migration, dendritic cells appeared to maintain an immature phenotype in the presence of fibronectin, expressed higher levels of endocytic receptors, and had a greater potential to induce T cell activation.

In 2011, Bechetoille et al. developed a dermal equivalent model of bovine collagen, chitosan, chondroitin sulfate, fibroblasts, and dermal macrophages derived from monocytes [104]. Macrophages, with the “classical” fusiform morphology, expressed CD14, CD163, and DC-SIGN/CD209 markers and produced large amounts of IL-10 in response to LPS, but little TNF. While LPS stimulated immune responses by interacting with the CD14 membrane receptor and induced the secretion of proinflammatory cytokines, such as TNF-*α*, IL-1, and IL-6, a stimulation of LPS macrophages in this *in vitro* model promoted their anti-inflammatory activity.

In 2014, van den Bogaard et. al developed a three-dimensional healthy *in vitro* skin substitute model in which they injected allogeneic healthy T lymphocytes into the dermal compartment. The authors demonstrated that the polarization of T cells towards a Th1 or Th17 phenotype, then injected in skin substitutes, induced the expression of molecular markers associated with psoriasis, although no hyperproliferation or acanthosis was observed. The inflammatory phenotype thus developed is similar to the psoriatic phenotype observed in human lesional plaques [105].

In another context, it has also been demonstrated that the generation of glycated cutaneous equivalents promoted the differentiation of monocytic cells towards a macrophage or dendritic cell phenotype. It would be interesting to characterize the influence of these differentiated cells on the modulation of the secretory profile in glycated substitutes [106] (Table 2).

## 5. Future Directions

The aim of this review was to analyze the importance of immune cells, and more particularly leukocyte cells, as neutrophils and macrophages in the pathophysiology of psoriasis. The emphasis is often on T cells, yet the influence of other immune cells on lymphocytes and keratinocytes must be better characterized. The specific roles of each cell types must be dissected to better understand the cellular hierarchy established in psoriasis. We also discussed the need to develop new *in vitro* models with a three-dimensional microenvironment and appropriate and relevant cellular components to better mimic the pathology and improve our understanding of it. Besides, it would also be advantageous to integrate other types of immune cells, such as dendritic cells, into human skin three-dimensional models, given their role in the presentation of antigens to T lymphocytes. Although there is a great temptation to develop a more complete model including immune, cutaneous, and endothelial cells to better reflect the actors involved in inflammation, it is often difficult to know if an observed effect is due to a single cell type or an interaction between anatomically close cells.

The development of complex three-dimensional models is an important issue for research in pharmacology. Today, most of the available models reproduce only very partially the *in vivo* situation because their architecture does not consider the complexity of the tissue interfaces and the vascular perfusion. Recently, some companies have begun to develop organs or tissues as relevant tools to repetitively assess the pharmacological action of drugs. For instance, Organovo, based in the United States, commercialized its first liver model in 2014, which incorporates hepatocytes, stellate cells, and endothelial cells, printed in a matrix. This model would be more discriminating than 2D cultures and would make it possible to mimic a patient's response to a drug [107]. It would be wise to focus on these *in vitro* models, given the ethical and financial constraints associated with the use of animal models.

Recently, new antipsoriatic therapies have emerged, such as apremilast (Otezla, Celgene), a phosphodiesterase 4 (PDE4) inhibitor, which is also the first oral anti-inflammatory treatment for psoriasis in more than 20 years. PDE4 inhibition would also be a potential target for systemic sclerosis, as its blockage decreased dermal fibrosis through the downregulation of profibrotic mediators from M2 macrophages [108].

Other promising drugs are currently being tested in phase III in the US, specifically targeting the inhibition of phospholipase A2 (PLA2), which controls the biosynthesis of inflammatory mediators, such as leukotrienes and prostaglandins. Other biological agents, such as etanercept (Enbrel, Amgen), infliximab (Remicade, Merck & Co./Janssen Biotech), and adalimumab (Humira, AbbVie), which are tumor necrosis factor (TNF) antagonists, are more commonly administered, although some side effects are frequently observed in patients (>10%): viral infection, dyspnea, migraine, and nausea. Since 2009, biological agents against psoriasis targeting IL-12, IL-17, and IL-23, cytokines that play a key role in inflammatory and immune responses, have been licensed on the market. Ustekinumab was the first drug specifically designed to suppress inflammation by targeting the signaling pathway of the cytokine family of interleukin-12 (IL-12) and interleukin-23 (IL-23).

Another therapeutic approach, the encapsulation of antipsoriatic agents in nanoparticles [109, 110], is currently under study with the aim of improving the efficacy, safety, and compliance of potential agents.

Advances in psoriasis research continue to lead to new therapeutic strategies that promise better management of this complex disease in the future. For sure, development of *in vitro* models and mouse models will help to revolutionize the care of psoriasis in the years to come.

## Figures and Tables

**Table 1:** The role of macrophages and neutrophils in different mouse models of psoriasis or other immune diseases.

*In vivo* models	Mouse model	Phenotype	Histopathological hallmarks	Role of macrophages	Role of neutrophils
Schon et al. [61]	Flaky skin	Psoriasiform skin lesions	Prominent infiltrate of neutrophils, and microabscesses within the hyperproliferative epidermis, hyperkeratosis, mixed inflammatory immune infiltrate	x	Yes
Wang et al. [44]	Hypomorphic PL/J CD18	Psoriasis-like skin inflammation	Abnormal keratinocyte proliferation/differentiation, subcorneal microabscesses, increased inflammatory infiltrate	Yes	Not causal for the maintenance of the skin inflammation
Stratis et al. [45]	K14-Cre-IKK2fl/fl	Inflammatory and hyperproliferative cutaneous phenotype	Hyperplastic epidermis with loss of the granular layer, focal parakeratosis, infiltration of the dermis with macrophages, T cells, mast cells, granulocytes and microabscesses	Yes	Not required for the development of the disease
Ward et al. [49]	KC-Tie2	Cutaneous psoriasiform phenotype	Acanthosis, increased CD4-positive T cells, epidermal CD8-positive T cells, dermal dendritic cells and macrophages	Yes	x
Sumida et al. [72]	Topical application model of imiquimod	Hyperplastic cutaneous epithelial-squamous phenotype	Epidermal proliferation, abnormal differentiation, epidermal accumulation of neutrophils in microabcesses, neoangiogenesis and infiltrates of immune cells	x	Yes
Keijsers et al. [69]	Leukotriene application Tape-stripping	Skin inflammation *in vivo* associated with the histopathology of psoriasis	Epidermal proliferation, influx of polymorphonuclear cells in the epidermis and dermis, followed by a mononuclear cell infiltrate	x	Yes
Leite Dantas et al. [54]	Doxycycline-inducible human TNF*α*–transgenic mouse	Inflammatory arthritis and psoriasis-like phenotype	Hyperproliferation and aberrant activation of keratinocytes, infiltration with Th1, Treg lymphocytes and macrophages	Yes	x
Morimura et al. [47]	Topical application model of imiquimod in CX3CR1-deficient mouse	Hyperplastic cutaneous epithelial-squamous phenotype	Epidermal proliferation, abnormal differentiation, epidermal accumulation of neutrophils in microabcesses, neoangiogenesis and infiltrates of immune cells	Yes	x
Zhang et al. [55]	K14-VEGF-A-transgenic mouse	Psoriasis-like chronic inflammatory skin disease	Epidermal hyperplasia, impaired epidermal differentiation, accumulation of dermal CD4 lymphocytes and epidermal CD8 lymphocytes	Yes	x

**(a):** 

3D *in vitro* models	Components	Support	Observed features
Dezutter-Dambuyant et al. [102]	Fibroblasts + keratinocytes + endothelialized cells + CD34-positive cells	Solid scaffold of bovine collagen, chitosan and chondroitin 4-6 sulfate	Differentiation of interstitial dendritic dells
Bechetoille et al. [104]	Fibroblasts + dermal macrophages derived from monocytes	Solid scaffold of bovine collagen, chitosan and chondroitin 4-6 sulfate	Display phagocytosis and remain responsive to LPS
Chau et al. [103]	Fibroblasts + keratinocytes + dendritic cells	Nondegradable microfibre scaffolds and a cell-laden gel	Able to migrate and remain responsive to stimulation with skin sensitizers
Pageon et al. [106]	Fibroblasts + keratinocytes + monocytes	AGE-modified collagen lattices	Differentiation of CD14+ monocytes into dendritic cells and macrophages

**Table:** (b) 3D models for psoriasis (without immune cells).

3D *in vitro* models	Components	Support	Observed features
Barker et al. [111]	Fibroblasts + keratinocytes isolated from human lesional skin	Collagen gels	Psoriasis-like phenotype
Tjabringa et al. [98]	Healthy keratinocytes + de-epidermized dermis + cytokines (IL-1*α*, TNF-*α*, IL-6 and IL-22)	De-epidermized dermis	Psoriasis-like phenotype
Jean et al. [97]	Psoriatic fibroblasts + psoriatic keratinocytes	Fibroblast-derived dermal matrix	Psoriasis-like phenotype
van den Bogaard et al. [105]	Healthy keratinocytes + activated CD4-positive T cells and Th1/Th17-polarized T cells	Decellularized deepidermized dermis	Psoriasis-like phenotype
